# Glial Cells in Amyotrophic Lateral Sclerosis

**DOI:** 10.1155/2011/718987

**Published:** 2011-06-07

**Authors:** Jurate Lasiene, Koji Yamanaka

**Affiliations:** ^1^Laboratory for Motor Neuron Disease, RIKEN Brain Science Institute, 2-1 Hirosawa, Wako-Shi, Saitama 351-0198, Japan; ^2^CREST, Japan Science and Technology Agency, Tokyo 102-0075, Japan; ^3^Department of State-of-the-Art and International Medicine, Graduate School of Medicine, Kyoto University, Kyoto 606-8501, Japan

## Abstract

Amyotrophic lateral sclerosis (ALS) is an adult motor neuron disease characterized by premature death of upper and lower motor neurons. Two percent of ALS cases are caused by the dominant mutations in the gene for superoxide dismutase 1 (SOD1) through a gain of toxic property of mutant protein. Genetic and chimeric mice studies using SOD1 models indicate that non-neuronal cells play important roles in neurodegeneration through non-cell autonomous mechanism. We review the contribution of each glial cell type in ALS pathology from studies of the rodent models and ALS patients. Astrogliosis and microgliosis are not only considerable hallmarks of the disease, but the intensity of microglial activation is correlated with severity of motor neuron damage in human ALS. The impaired astrocytic functions such as clearance of extracellular glutamate and release of neurotrophic factors are implicated in disease. Further, the damage within astrocytes and microglia is involved in accelerated disease progression. Finally, other glial cells such as NG2 cells, oligodendrocytes and Schwann cells are under the investigation to determine their contribution in ALS. Accumulating knowledge of active role of glial cells in the disease should be carefully applied to understanding of the sporadic ALS and development of therapy targeted for glial cells.

## 1. Introduction

Amyotrophic lateral sclerosis (ALS) is characterized by premature death of upper and lower motor neurons starting in adulthood. The pathology of ALS is characterized by abnormal accumulation of insoluble and misfolded proteins in degenerating motor neurons. Neuronal death results in progressive paralysis, which typically is fatal 2–5 years after the onset due to respiratory failure. Ten percent of ALS cases are inherited, while the rest are considered sporadic and the cause has not been discovered yet. Twenty percent of inherited ALS cases are caused by mutations in the gene encoding for superoxide dismutase 1 (SOD1) [1, 2]. SOD1, a ubiquitously expressed enzyme, catalytically converts reactive superoxide to oxygen and hydrogen peroxide. It is now recognized that all different mutations of SOD1 gene (both enzymatically active and inactive mutants) uniformly cause toxicity in cells not by loss but rather by gain of function where accumulation of protein in neurons and glia causes toxicity. However, the exact mechanism and nature of toxicity are still unknown [3, 4]. Currently, numerous mechanisms of toxicity have been proposed that could mediate pathology in mutant SOD1-mediated ALS. The most important mechanisms are thought to be excitotoxicity from glutamate, failure of protein degradation machinery, ER stress, damage to mitochondria, superoxide generation through neuroinflammation, axonal transport disruption, and spinal capillary microhemorrhages [5–11]. There is good evidence for all of these mechanisms to be at play, and most likely it is a combination of different events that contribute to the overall development of ALS pathology.

The discovery of SOD1 mutations led to the development of animal models that recapitulate ALS-like disease. Overproduction of mutated human SOD1 protein in these mouse models leads to a progressive neurodegenerative disease that closely resembles human pathology with a selective motor neuron death and gliosis accompanied by accumulation of misfolded proteins [12]. 

The selective death of motor neurons initially led the researchers to believe that cell autonomous mechanisms were at play. However, genetic and chimeric mice studies indicate that non-cell autonomous processes might underlie motor neuron loss in these rodent examples and hence potentially in ALS. First, when expression of SOD1 mutations was restricted to either motor neurons or astrocytes, but not in both simultaneously, it did not lead to the development of ALS [13–15]. A recent study succeeded to produce very late onset disease in mice when mutant is expressed in all neurons [16] however, the severity and rapidity of disease progression of the mice were much more modest compared with mice expressing the same mutant gene ubiquitously. Second, wild-type neurons, in chimeric mice with both wild-type and mutant SOD1-expressing cells, acquired an ALS phenotype when surrounded by glial cells bearing SOD1 mutation [17]. Finally, removal of mutant SOD1 expression in either astrocytes or microglia using floxed SOD1 gene excised by Cre recombinase slowed the disease progression and extended life expectancy [18–21]. Immunohistological studies also show glial cell involvement in ALS pathology where astrogliosis and microgliosis are considerable hallmarks of the disease [22, 23]. Among the non-neuronal cell types, a role of glial cells in ALS has been most intensively investigated. Here, we review the contribution of each glial cell type in ALS pathology from studies of the rodent models and ALS patients.

## 2. Microglia

Microglia, derived from the hematopoietic cell lineage, are generally considered as the primary immune cells of the central nervous system. Microglia are distributed throughout the CNS and are continuously surveying the environment with mobile arborizations of cell processes. Under normal conditions, these cells have been called “resting microglia,” the term recently questioned due to recognition that these cells are actually continuously providing surveillance in the nervous system [24]. They sense and react to many types of damage, such as microbial infection, serum microhemorrhage of blood vessels, immunoglobulin-antigen complexes, and abnormal proteins produced in the neurodegenerative diseases. In response to such stimuli, microglia change their morphology from ramified to amoeboid form, migrate to the damaged cells, and subsequently clear the debris of the dead cells. Through such processes, microglia release reactive oxygen species, proinflammatory cytokines, complement factors, and neurotoxic molecules, leading to further neuronal dysfunction and death, which is a vicious cycle called as neuroinflammation [25]. 

Gliosis has long been known as a component of ALS pathology, with microgliosis recognized in the past 20 years [26, 27]. Further, recent work using positron emission tomography provided direct evidence of widespread microglial activation in the brains of living ALS patients [28]. The intensity of microglial activation was correlated with severity of upper motor neuron damage, suggesting an active involvement of microglial activation in the disease. Extensive microgliosis and inflammation accompanied by elevated level of proinflammatory cytokines are reproducibly demonstrated in the lesion of mutant SOD1 transgenic mice [22, 23]. Molecules released from activated microglia include proinflammatory cytokines (tumor necrosis factor-*α*, interleukin-1*β*, interleukin-12, interferon-*γ*, and others), reactive oxygen species (superoxide, nitric oxide, and its derivatives), chemokines and mitogenic factors (monocyte chemoattractant protein 1, macrophage colony stimulating factor), anti-inflammatory cytokines (tumor growth factor-*β*), and neurotrophic factors (IGF-1: insulin-like growth factor-1) [29–33]. 

A detrimental role of mutant SOD1 in microglia was first demonstrated in a cell culture system. Mutant SOD1-expressing microglia released higher levels of Tumor Necrosis Factor-*α* and Interleukin-6 in comparison to the wild-type microglia when stimulated with lipopolysaccharide (LPS) [34]. Non-cell autonomous effects of mutant microglia were further confirmed by showing reduced survival rates of the primary cultured motor neurons when cocultured with mutant microglia [35]. 

The active role of microglia in disease progression was demonstrated by three independent experiments. Selective reduction of mutant SOD1 from microglia/macrophages in mice using Cre-Lox system demonstrated that mutant toxicity within microglia slowed disease progression in SOD1^G37R^ [19] and SOD1^G85R^ mice [20]. A complimentary approach using replacing microglia/macrophage via bone marrow transplantation reached the same conclusion and demonstrated that wild-type microglia/macrophage slowed disease progression in SOD1^G93A^ mice [36]. 

The identity of factors that activate microglia has been explored. The innate immune system is the first line of defense against invading pathogens which are recognized mainly through toll-like receptors (TLRs). Elevated level of innate immune receptors such as TLR2 and CD14 was recorded in mutant SOD1 mice. Further, bone marrow deficient of MyD88 (myeloid differentiation factor 88), essential adaptor protein to transmit most of TLR signaling, accelerated disease progression in SOD1^G37R^ mice [37]. This outcome is likely related to an effect of irradiation in the process of chimeric mice generation [38, 39]. Indeed, gene deletion of MyD88 had no effect on disease course of SOD1^G37R^ mice [37]. To date, factors known to be released from damaged neurons in ALS models are ATP and extracellular SOD1, which activate microglia *in vitro* through purinergic receptors and CD14, respectively [40, 41]. Other factors central to damaged motor neuron-microglial communication and the role of innate immune system in ALS should be explored further.

In contrast to innate immune system, the role of acquired immunity in ALS has recently been extensively investigated. The presence of T lymphocyte in ALS mouse models as well as sporadic ALS patients [42] suggested the involvement of acquired immunity in ALS. Genetic ablation of CD4+T cells or functional T cells (RAG2 gene deletion) accelerated disease progression in ALS mice [43, 44]. In those studies, presence of CD4+T cells was considered to stabilize microglial activation status with decreased level of proinflammatory cytokines and increase of neurotrophic factor, IGF-1. Another recent study showed that transferring activated CD4+CD25+ cells extended life span of mutant SOD1 mice [45]. These studies support a protective role of specific population of T lymphocytes through controlled microglial activation.

## 3. Astrocytes

Astrocytes have many important functions in maintaining and nourishing central nervous system (CNS) neurons. One of the main important functions is to maintain low extracellular concentration of glutamate. Astrocytes clear the excess of glutamate neurotransmitter from the synaptic clefts mostly by employing EAAT2/GLT-1 glutamate transporter. Overabundance of glutamate leads to neuronal excitotoxicity due to excessive neuronal firing and corresponding increased influx of calcium. Accumulating evidence demonstrates that astrocytic function of clearing glutamate is impaired due to a loss of EAAT2/GLT1 transporter in sporadic and familial ALS human cases as well as SOD1 mouse models [5, 46, 47]. Riluzole, a pharmacological agent that reduces glutamate release from nerve terminals and is the only currently clinically approved drug for ALS, supports the role of excitotoxicity in ALS. Finally, mutant SOD1 astrocytes secrete factors that lower the expression of the GluR2 glutamate receptor subunit, which results in more Ca^2+^ permeable AMPA receptors in motor neurons. However, if mutant SOD1 astrocytes are not present, mutant SOD1 in motor neurons does not have the same effect, which again shows non-cell autonomous death mechanisms in ALS [48].

A second role that can be attributed to deleterious astrocyte behavior in ALS is an insufficient release of neurotrophic factors that are important in maintaining neuronal health. Glial-derived neurotrophic factor, brain-derived neurotrophic factor, ciliary neurotrophic factor, and vascular endothelial growth factor are all released by astrocytes and can rescue motor neurons [49, 50]. A loss of neurotrophins if not directly, then indirectly, might be a cause of neuronal death. In addition to neurotrophins, astrocytes may release hazardous factors. *In vitro* studies confirm that factors released by SOD1 astrocytes in culture media can induce apoptosis in motor neuron cultures. One of the identified toxic factors is neurotrophic growth factor (NGF) [51]. Similarly, wild-type embryonic stem (ES) cell-derived motor neurons co-cultured with mutant SOD1-expressing GFAP positive astrocytes are induced to degenerate and die indicating non-cell autonomous degeneration mechanism [52–55].

The role of astrocytes in ALS pathology has been widely recognized and appreciated. Astrocytes have become an interesting therapeutic target, and more new studies of intervention are coming to light. The transcription factor, Nrf2, regulates the expression of genes containing antioxidant response element (ARE), which are preferentially activated in astrocytes. An attempt to activate ARE/Nrf2 in astrocytes has been successful in protecting neighboring neurons *in vitro*, and extends the survival in ALS mice [56].

On the other hand, in one study where proliferating astrocytes were a target of selective ablation, neither onset nor progression of disease was affected in mutant SOD1 mice [57]. It has also been shown that ablation of astrocytes in injury models does not help the outcome as the astrocytes might play a protective role [58]. In contrast, transplantation of healthy glial precursor cells, which later differentiated into astrocytes, proved to be neuroprotective and extended survival time in mutant SOD1 model [59]. The benefit of transplanting healthy glial cells is in accordance with works in which Cre-mediated ablation of mutant SOD1 transgenes selectively from GFAP-positive astrocytes extended lifespan of ALS mice [18, 21].

## 4. Oligodendrocytes and NG2 Cells

Microglia and astrocytes have been recognized as major players in ALS disease. However, other glial cells such as NG2 cells (sometimes called synantocytes or pericytes) and oligodendrocytes have not been investigated to a large extent. There are very few reports suggesting that these glial cells might be involved in ALS pathogenesis. A study of human ALS postmortem tissue showed diffuse myelin pallor in the anterolateral columns associated with microglial infiltration and loss in number of small fibers most likely due to intrinsic spinal cord lesions [60]. A later study examined myelin state in ALS in more detail and they found that myelin abnormalities such as a loss of compact myelin, lamellae detachment, and a decrease in lipid content were evident in presymptomatic cords. More pronounced morphological and biochemical myelin degeneration was evident in fully symptomatic stages of mutant SOD1 rats [61]. It is too early to speculate whether oligodendrocytes or myelin sheaths have any role in ALS disease onset and progression, but the few studies that examined the issue suggest that it might be an interesting target for further study. In contrast, the most recent study employing chimeric mice suggested that oligodendrocytes might not be an important element in the disease pathology. The researchers examined chimeric mice whose all motor neurons and oligodendrocytes expressed high levels of mutant SOD1. Disease onset was substantially delayed in the mice suggesting non-cell autonomous mechanism where cell types other than motor neurons and oligodendrocytes must be major contributors to ALS disease onset and perhaps progression [62].

NG2 cells (marked by nerve-glia factor 2 proteoglycan antibody) have been very little examined in the context of ALS pathology. NG2 cells are one of the first cells to respond to any changes in CNS environment. They assume activated morphology and start dividing due to any insults or disturbances to the CNS where they contribute to changing cellular environment with producing new astrocytes, oligodendrocytes, and, in some areas of the CNS, neurons. The first report established an increased cell division associated with the ALS disease progression and noticed that a percentage of NG2 cells become astrocytes most likely due to proinflammatory cytokine signaling [63]. However, a very recent study demonstrated that the majority of NG2 cells remain committed to an oligodendrocyte lineage in the adult wild-type mice as well as symptomatic SOD1^G93A^ mice, suggesting that NG2 cells do not play a major role in astrogliosis [64]. NG2 cells might participate not only in contributing to astrogliosis but also in other yet undiscovered ways. Another reason why it is important to understand NG2 cell role in ALS is that due to a regenerative capacity of these cells they might be mobilized to generate cells, and secrete factors conducive to beneficial ALS outcome.

## 5. Schwann Cells

Schwann cells—peripheral myelin generating cells—are closely associated with motor neuron axons and aid the axonal development and regeneration. So far, very little is known about Schwann cell involvement in ALS pathology. Studies of human ALS show peripheral myelin changes along the motor neuron axons which are most likely due to axonal degeneration [65]. Recent studies show limited or unexpected Schwann cell involvement in the disease progression. One study expressed mutant SOD1^G93A^ transgene only in protein zero (P0) positive Schwann cells and these mice were identical to control animals with no changes to locomotion, neuronal loss, or axonal degeneration [66]. The study demonstrates the lack of specific causal involvement of myelinating P0 Schwann cells in the ALS disease onset or progression. A second study used a different approach, and, instead of inducing higher synthesis of SOD1, they removed mutant SOD1^G37R^ from Schwann cells using Cre-mediated gene excision. Surprisingly, the authors discovered that even though disease onset was not altered, the disease progression was dramatically accelerated suggesting a connection between disease progression in ALS and a protective effect of mutant SOD1^G37R^ in Schwann cells. Finally, they observed that reduced mutant SOD1 expression was associated with diminished levels of insulin-like growth factor 1 [67]. A close relationship between Schwann cells and motor neuron axons warrants more studies to help us better understand the pathology of ALS.

## 6. Conclusion and Perspective

Active contributions of glial cells in ALS pathology have recently been extensively demonstrated as reviewed here. Finally, it should be well considered whether translating the research results using SOD1 rodent models into understanding and development of treatment for sporadic ALS. To date, several clinical trials using drugs targeting glial cells were designed for sporadic ALS patients. These drugs were proved to have effect on mutant SOD1 mice. Antibiotics, minocycline and cyclooxygenase 2 inhibitor, celecoxib, were effective to extend the survival for mutant SOD1 mice [68–71]. However, ALS patients did not tolerate minocycline well, and there was no evidence demonstrating slowing of the disease in the phase III clinical trial [72]. A recent mouse study, in which minocycline was administered after disease onset exacerbated neuroinflammation, explains the failure of human clinical trial [73]. Similarly, clinical trial of celecoxib for sporadic ALS patients showed no effect, although the dose of celecoxib used for trial did not decrease the level of Prostaglandin E(2) in CSF [74]. Failure to translate results of rodent models to sporadic human patients was attributed to several reasons [75]. 

First, in many preclinical studies, the drugs were administered to animals before onset. However, this is not the case for sporadic ALS patients, since human patients are treated after the diagnosis. Second, in many cases the drug effects aiming to extend the survival time of mice were modest, with cohort sizes that were not sufficient enough. Adequate cohort size as well as the timing of initiating drug treatment should be carefully considered in the rodent studies [76]. Third, the disease mechanism of mutant SOD1-mediated familial ALS could be different from sporadic ALS. Recent discovery of new genes, TDP-43, FUS, responsible for ALS has provided new opportunity to the development of new animal models [77, 78]. New rodent models useful for testing new candidate drugs are awaited. Lastly, the molecules misregulated in the glial cells in mutant SOD1-mediated ALS should be re-evaluated in human sporadic ALS cases. 

Controlling neuroinflammation and communication to immune system have also been the focus in other neurodegenerative diseases including Alzheimer's and Parkinson's diseases [79, 80]. Further understanding of molecular pathology within glial cells will contribute to developing therapies that will slow ALS disease progression benefiting sporadic and familiar ALS patients.

## References

[1] Rosen DR, Siddique T, Patterson D (1993). Mutations in Cu/Zn superoxide dismutase gene are associated with familial amyotrophic lateral sclerosis. *Nature*.

[2] Pasinelli P, Brown RH (2006). Molecular biology of amyotrophic lateral sclerosis: insights from genetics. *Nature Reviews Neuroscience*.

[3] Bruijn LI, Miller TM, Cleveland DW (2004). Unraveling the mechanisms involved in motor neuron degeneration in ALS. *Annual Review of Neuroscience*.

[4] Turner BJ, Talbot K (2008). Transgenics, toxicity and therapeutics in rodent models of mutant SOD1-mediated familial ALS. *Progress in Neurobiology*.

[5] Rothstein JD, van Kammen M, Levey AI, Martin LJ, Kuncl RW (1995). Selective loss of glial glutamate transporter GLT-1 amyotrophic lateral sclerosis. *Annals of Neurology*.

[6] Yang Y, Gozen O, Watkins A (2009). Presynaptic regulation of astroglial excitatory neurotransmitter transporter GLT1. *Neuron*.

[7] Kikuchi H, Almer G, Yamashita S (2006). Spinal cord endoplasmic reticulum stress associated with a microsomal accumulation of mutant superoxide dismutase-1 in an ALS model. *Proceedings of the National Academy of Sciences of the United States of America*.

[8] Liu J, Lillo C, Jonsson PA (2004). Toxicity of familial ALS-linked SOD1 mutants from selective recruitment to spinal mitochondria. *Neuron*.

[9] Harraz MM, Marden JJ, Zhou W (2008). SOD1 mutations disrupt redox-sensitive Rac regulation of NADPH oxidase in a familial ALS model. *Journal of Clinical Investigation*.

[10] Williamson TL, Cleveland DW (1999). Slowing of axonal transport is a very early event in the toxicity of ALS-linked SOD1 mutants to motor neurons. *Nature Neuroscience*.

[11] Zhong Z, Deane R, Ali Z (2008). ALS-causing SOD1 mutants generate vascular changes prior to motor neuron degeneration. *Nature Neuroscience*.

[12] Gurney ME, Pu H, Chiu AY (1994). Motor neuron degeneration in mice that express a human Cu,Zn superoxide dismutase mutation. *Science*.

[13] Lino MM, Schneider C, Caroni P (2002). Accumulation of SOD1 mutants in postnatal motoneurons does not cause motoneuron pathology or motoneuron disease. *Journal of Neuroscience*.

[14] Pramatarova A, Laganière J, Roussel J, Brisebois K, Rouleau GA (2001). Neuron-specific expression of mutant superoxide dismutase 1 in transgenic mice does not lead to motor impairment. *Journal of Neuroscience*.

[15] Gong YH, Parsadanian AS, Andreeva A, Snider WD, Elliott JL (2000). Restricted expression of G86R Cu/Zn superoxide dismutase in astrocytes results in astrocytosis but does not cause motoneuron degeneration. *Journal of Neuroscience*.

[16] Jaarsma D, Teuling E, Haasdijk ED, de Zeeuw CI, Hoogenraad CC (2008). Neuron-specific expression of mutant superoxide dismutase is sufficient to induce amyotrophic lateral sclerosis in transgenic mice. *Journal of Neuroscience*.

[17] Clement AM, Nguyen MD, Roberts EA (2003). Wild-type nonneuronal cells extend survival of SOD1 mutant motor neurons in ALS mice. *Science*.

[18] Yamanaka K, Chun SJ, Boillee S (2008). Astrocytes as determinants of disease progression in inherited amyotrophic lateral sclerosis. *Nature Neuroscience*.

[19] Boillée S, Yamanaka K, Lobsiger CS (2006). Onset and progression in inherited ALS determined by motor neurons and microglia. *Science*.

[20] Wang L, Sharma K, Grisotti G, Roos RP (2009). The effect of mutant SOD1 dismutase activity on non-cell autonomous degeneration in familial amyotrophic lateral sclerosis. *Neurobiology of Disease*.

[21] Wang L, Gutmann DH, Roos RP (2011). Astrocyte loss of mutant SOD1 delays ALS disease onset and progression in G85R transgenic mice. *Human Molecular Genetics*.

[22] Hall ED, Oostveen JA, Gurney ME (1998). Relationship of microglial and astrocytic activation to disease onset and progression in a transgenic model of familial ALS. *GLIA*.

[23] Alexianu ME, Kozovska M, Appel SH (2001). Immune reactivity in a mouse model of familial ALS correlates with disease progression. *Neurology*.

[24] Hanisch UK, Kettenmann H (2007). Microglia: active sensor and versatile effector cells in the normal and pathologic brain. *Nature Neuroscience*.

[25] Heneka MT, Rodríguez JJ, Verkhratsky A (2010). Neuroglia in neurodegeneration. *Brain Research Reviews*.

[26] McGeer PL, Itagaki S, Tago H, McGeer EG (1987). Reactive microglia in patients with senile dementia of the Alzheimer type are positive for the histocompatibility glycoprotein HLA-DR. *Neuroscience Letters*.

[27] Engelhardt JI, Appel SH (1990). IgG reactivity in the spinal cord and motor cortex in amyotrophic lateral sclerosis. *Archives of Neurology*.

[28] Turner MR, Cagnin A, Turkheimer FE (2004). Evidence of widespread cerebral microglial activation in amyotrophic lateral sclerosis: an [11C](R)-PK11195 positron emission tomography study. *Neurobiology of Disease*.

[29] Almer G, Vukosavic S, Romero N, Przedborski S (1999). Inducible nitric oxide synthase up-regulation in a transgenic mouse model of familial amyotrophic lateral sclerosis. *Journal of Neurochemistry*.

[30] Elliott JL (2001). Cytokine upregulation in a murine model of familial amyotrophic lateral sclerosis. *Molecular Brain Research*.

[31] Yoshihara T, Ishigaki S, Yamamoto M (2002). Differential expression of inflammation- and apoptosis-related genes in spinal cords of a mutant SOD1 transgenic mouse model of familial amyotrophic lateral sclerosis. *Journal of Neurochemistry*.

[32] Hensley K, Fedynyshyn J, Ferrell S (2003). Message and protein-level elevation of tumor necrosis factor *α* (TNF*α*) and TNF*α*-modulating cytokines in spinal cords of the G93A-SOD1 mouse model for amyotrophic lateral sclerosis. *Neurobiology of Disease*.

[33] Hensley K, Floyd RA, Gordon B (2002). Temporal patterns of cytokine and apoptosis-related gene expression in spinal cords of the G93A-SOD1 mouse model of amyotrophic lateral sclerosis. *Journal of Neurochemistry*.

[34] Weydt P, Yuen EC, Ransom BR, Möller T (2004). Increased cytotoxic potential of microglia from ALS-transgenic mice. *GLIA*.

[35] Xiao Q, Zhao W, Beers DR (2007). Mutant SOD1 microglia are more neurotoxic relative to wild-type microglia. *Journal of Neurochemistry*.

[36] Beers DR, Henkel JS, Xiao Q (2006). Wild-type microglia extend survival in PU.1 knockout mice with familial amyotrophic lateral sclerosis. *Proceedings of the National Academy of Sciences of the United States of America*.

[37] Kang J, Rivest S (2007). MyD88-deficient bone marrow cells accelerate onset and reduce survival in a mouse model of amyotrophic lateral sclerosis. *Journal of Cell Biology*.

[38] Ajami B, Bennett JL, Krieger C, Tetzlaff W, Rossi FMV (2007). Local self-renewal can sustain CNS microglia maintenance and function throughout adult life. *Nature Neuroscience*.

[39] Mildner A, Schmidt H, Nitsche M (2007). Microglia in the adult brain arise from Ly-6ChiCCR2+ monocytes only under defined host conditions. *Nature Neuroscience*.

[40] D’Ambrosi N, Finocchi P, Apolloni S (2009). The proinflammatory action of microglial P2 receptors is enhanced in SOD1 models for amyotrophic lateral sclerosis. *Journal of Immunology*.

[41] Zhao W, Beers DR, Henkel JS (2010). Extracellular mutant SOD1 induces microglial-mediated motoneuron injury. *GLIA*.

[42] Engelhardt JI, Tajti J, Appel SH (1993). Lymphocytic infiltrates in the spinal cord in amyotrophic lateral sclerosis. *Archives of Neurology*.

[43] Beers DR, Henkel JS, Zhao W, Wang J, Appel SH (2008). CD4+ T cells support glial neuroprotection, slow disease progression, and modify glial morphology in an animal model of inherited ALS. *Proceedings of the National Academy of Sciences of the United States of America*.

[44] Chiu IM, Chen A, Zheng Y (2008). T lymphocytes potentiate endogenous neuroprotective inflammation in a mouse model of ALS. *Proceedings of the National Academy of Sciences of the United States of America*.

[45] Banerjee R, Mosley RL, Reynolds AD (2008). Adaptive immune neuroprotection in G93A-SOD1 amyotrophic lateral sclerosis mice. *PLoS ONE*.

[46] Fray AE, Ince PG, Banner SJ (1998). The expression of the glial glutamate transporter protein EAAT2 in motor neuron disease: an immunohistochemical study. *European Journal of Neuroscience*.

[47] Howland DS, Liu J, She Y (2002). Focal loss of the glutamate transporter EAAT2 in a transgenic rat model of SOD1 mutant-mediated amyotrophic lateral sclerosis (ALS). *Proceedings of the National Academy of Sciences of the United States of America*.

[48] van Damme P, Bogaert E, Dewil M (2007). Astrocytes regulate GluR2 expression in motor neurons and their vulnerability to excitotoxicity. *Proceedings of the National Academy of Sciences of the United States of America*.

[49] Ekestern E (2004). Neurotrophic factors and amyotrophic lateral sclerosis. *Neurodegenerative Diseases*.

[50] Dewil M, Lambrechts D, Sciot R (2007). Vascular endothelial growth factor counteracts the loss of phospho-Akt preceding motor neurone degeneration in amyotrophic lateral sclerosis. *Neuropathology and Applied Neurobiology*.

[51] Pehar M, Cassina P, Vargas MR (2004). Astrocytic production of nerve growth factor in motor neuron apoptosis: implications for amyotrophic lateral sclerosis. *Journal of Neurochemistry*.

[52] Nagai M, Re DB, Nagata T (2007). Astrocytes expressing ALS-linked mutated SOD1 release factors selectively toxic to motor neurons. *Nature Neuroscience*.

[53] Di Giorgio FP, Boulting GL, Bobrowicz S, Eggan KC (2008). Human embryonic stem cell-derived motor neurons are sensitive to the toxic effect of glial cells carrying an ALS-causing mutation. *Cell Stem Cell*.

[54] Di Giorgio FP, Carrasco MA, Siao MC, Maniatis T, Eggan K (2007). Non-cell autonomous effect of glia on motor neurons in an embryonic stem cell-based ALS model. *Nature Neuroscience*.

[55] Marchetto MCN, Muotri AR, Mu Y, Smith AM, Cezar GG, Gage FH (2008). Non-cell-autonomous effect of human SOD1 G37R astrocytes on motor neurons derived from human embryonic stem cells. *Cell Stem Cell*.

[56] Vargas MR, Johnson DA, Sirkis DW, Messing A, Johnson JA (2008). Nrf2 activation in astrocytes protects against neurodegeneration in mouse models of familial amyotrophic lateral sclerosis. *Journal of Neuroscience*.

[57] Lepore AC, Dejea C, Carmen J (2008). Selective ablation of proliferating astrocytes does not affect disease outcome in either acute or chronic models of motor neuron degeneration. *Experimental Neurology*.

[58] Faulkner JR, Herrmann JE, Woo MJ, Tansey KE, Doan NB, Sofroniew MV (2004). Reactive astrocytes protect tissue and preserve function after spinal cord injury. *Journal of Neuroscience*.

[59] Lepore AC, Rauck B, Dejea C (2008). Focal transplantation-based astrocyte replacement is neuroprotective in a model of motor neuron disease. *Nature Neuroscience*.

[60] Hayashi S, Sakurai A, Amari M, Okamoto K (2001). Pathological study of the diffuse myelin pallor in the anterolateral columns of the spinal cord in amyotrophic lateral sclerosis. *Journal of the Neurological Sciences*.

[61] Niebroj-Dobosz I, Rafałowska J, Fidziańska A, Gadamski R, Grieb P (2007). Myelin composition of spinal cord in a model of amyotrophic lateral sclerosis (ALS) in SOD1G93A transgenic rats. *Folia Neuropathologica*.

[62] Yamanaka K, Boillee S, Roberts EA (2008). Mutant SOD1 in cell types other than motor neurons and oligodendrocytes accelerates onset of disease in ALS mice. *Proceedings of the National Academy of Sciences of the United States of America*.

[63] Magnus T, Carmen J, Deleon J (2008). Adult glial precursor proliferation in mutant SOD1G93A mice. *GLIA*.

[64] Kang SH, Fukaya M, Yang JK, Rothstein JD, Bergles DE (2010). NG2+ CNS glial progenitors remain committed to the oligodendrocyte lineage in postnatal life and following neurodegeneration. *Neuron*.

[65] Perrie WT, Lee GT, Curtis EM, Sparke J, Buller JR, Rossi ML (1993). Changes in the myelinated axons of femoral nerve in amyotrophic lateral sclerosis. *Journal of Neural Transmission, Supplement*.

[66] Turner BJ, Ackerley S, Davies KE, Talbot K (2009). Dismutase-competent SOD1 mutant accumulation in myelinating Schwann cells is not detrimental to normal or transgenic ALS model mice. *Human Molecular Genetics*.

[67] Lobsiger CS, Boillee S, McAlonis-Downes M (2009). Schwann cells expressing dismutase active mutant SOD1 unexpectedly slow disease progression in ALS mice. *Proceedings of the National Academy of Sciences of the United States of America*.

[68] Kriz J, Nguyen MD, Julien JP (2002). Minocycline slows disease progression in a mouse model of amyotrophic lateral sclerosis. *Neurobiology of Disease*.

[69] Drachman DB, Frank K, Dykes-Hoberg M (2002). Cyclooxygenase 2 inhibition protects motor neurons and prolongs survival in a transgenic mouse model of ALS. *Annals of Neurology*.

[70] Zhu S, Stavrovskaya IG, Drozda M (2002). Minocycline inhibits cytochrome c release and delays progression of amyotrophic lateral sclerosis in mice. *Nature*.

[71] van den Bosch L, Tilkin P, Lemmens G, Robberecht W (2002). Minocycline delays disease onset and mortality in a transgenic model of ALS. *NeuroReport*.

[72] Gordon PH, Moore DH, Miller RG (2007). Efficacy of minocycline in patients with amyotrophic lateral sclerosis: a phase III randomised trial. *Lancet Neurology*.

[73] Keller AF, Gravel M, Kriz J (2011). Treatment with minocycline after disease onset alters astrocyte reactivity and increases microgliosis in SOD1 mutant mice. *Experimental Neurology*.

[74] Cudkowicz ME, Shefner JM, Schoenfeld DA (2006). Trial of celecoxib in amyotrophic lateral sclerosis. *Annals of Neurology*.

[75] Benatar M (2007). Lost in translation: treatment trials in the SOD1 mouse and in human ALS. *Neurobiology of Disease*.

[76] Ludolph AC, Bendotti C, Blaugrund E (2010). Guidelines for preclinical animal research in ALS/MND: a consensus meeting. *Amyotrophic Lateral Sclerosis*.

[77] Pesiridis GS, Lee VM, Trojanowski JQ (2009). Mutations in TDP-43 link glycine-rich domain functions to amyotrophic lateral sclerosis. *Human Molecular Genetics*.

[78] Lagier-Tourenne C, Polymenidou M, Cleveland DW (2010). TDP-43 and FUS/TLS: emerging roles in RNA processing and neurodegeneration. *Human Molecular Genetics*.

[79] Hirsch EC, Hunot S (2009). Neuroinflammation in Parkinson’s disease: a target for neuroprotection?. *Lancet Neurology*.

[80] Lucin KM, Wyss-Coray T (2009). Immune activation in brain aging and neurodegeneration: too much or too little?. *Neuron*.

